# Iterated cross validation method for prediction of survival in diffuse large B-cell lymphoma for small size dataset

**DOI:** 10.1038/s41598-023-28394-6

**Published:** 2023-01-25

**Authors:** Chin-Chuan Chang, Chien-Hua Chen, Jer-Guang Hsieh, Jyh-Horng Jeng

**Affiliations:** 1 Department of Electrical Engineering, I-Shou University, Kaohsiung, 84001 Taiwan; 2 Department of Nuclear Medicine, Kaohsiung Medical University Hospital, Kaohsiung, 80756 Taiwan; 3 School of Medicine, College of Medicine, Kaohsiung Medical University, Kaohsiung, 80756 Taiwan; 4 Department of Emergency Medicine, Kaohsiung Municipal United Hospital, Kaohsiung, 80457 Taiwan; 5 Department of Information Engineering, I-Shou University, Kaohsiung, 84001 Taiwan

**Keywords:** Diseases, Medical research

## Abstract

Efforts have been made to improve the risk stratification model for patients with diffuse large B-cell lymphoma (DLBCL). This study aimed to evaluate the disease prognosis using machine learning models with iterated cross validation (CV) method. A total of 122 patients with pathologically confirmed DLBCL and receiving rituximab-containing chemotherapy were enrolled. Contributions of clinical, laboratory, and metabolic imaging parameters from fluorine-18 fluorodeoxyglucose (FDG) positron emission tomography/computed tomography (PET/CT) scans to the prognosis were evaluated using five regression models, namely logistic regression, random forest, support vector classifier (SVC), deep neural network (DNN), and fuzzy neural network models. Binary classification predictions for 3-year progression free survival (PFS) and 3-year overall survival (OS) were conducted. The 10-iterated fivefold CV with shuffling process was conducted to predict the capability of learning machines. The median PFS and OS were 41.0 and 43.6 months, respectively. Two indicators were found to be independent predictors for prognosis: international prognostic index and total metabolic tumor volume (MTVsum) from FDG PET/CT. For PFS, SVC and DNN (both with accuracy 71%) have the best predictive results, of which outperformed other algorithms. For OS, the DNN has the best predictive result (accuracy 76%). Using clinical and metabolic parameters as input variables, the machine learning methods with iterated CV method add the predictive values for PFS and OS evaluation in DLBCL patients.

## Introduction

Diffuse large B-cell lymphoma (DLBCL) is the most common type of lymphoma and accounts for approximately one-third of non-Hodgkin’s lymphoma^1^. The age-standardized rate (per 100,000) for male and female in Taiwan was only 3.44 and 2.54, respectively^1^. The causes of DLBCL are not well understood. The DLBCL can arise in any part of the body and is often an aggressive type of malignancy. Although it usually arises from the normal B lymphocytes, it can also represent a malignant transformation from other types of B-cell lymphoma. Typically, the first sign of this disease is the rapidly growing tissue or mass sometimes associated with the B symptoms (e.g., fever, weight loss, and night sweats). The diagnosis of DLBCL is made pathologically of the biopsied tissue. There are several subtypes of DLBCL identified which differ in their clinical presentations, symptoms, aggressiveness, morphology, immunophenotypes, gene expression, and the prognosis^2^. Because of its aggressiveness with complex outcomes due to the heterogeneous entity, complete pretreatment evaluation including prognostic classification is important for the disease management.

In the past 20 years, the international prognostic index (IPI) has been one of the most useful tools to evaluate the prognosis in patients with aggressive NHL including DLBCL. In the evaluation of IPI, one point is assigned for each of the following risk factors: (1) age over 60 years, (2) Stage III or IV disease, (3) elevated level of serum lactate dehydrogenase (LDH), (4) Eastern Cooperative Oncology Group (ECOG) performance status of 2–4, and (5) more than 1 extra-nodal site involvement of disease (i.e., site other than lymph nodes, spleen, thymus, and the pharyngeal lymphatic ring). The 5-year overall survival (OS) for low risk (IPI: 0–1), low-intermediate risk (IPI: 2), high-intermediate risk (IPI: 3), and high risk (IPI: 4–5) is 73%, 51%, 43%, and 26%, respectively^3^.

Since the addition of rituximab (an immunoglobulin G1 monoclonal antibody against B-lymphocyte antigen CD20) into the first-line chemotherapy (i.e., cyclophosphamide, doxorubicine, vincristine and prednisone) (R-CHOP), the 5-year survival rate of DLBCL has been improved^4^. However, patients with the same IPI score may still suffer from different outcomes either due to early relapse or refractory disease. Great efforts have been made to improve the evaluation models for risk stratification^5–8^, and more reliable prognostic predictors are pressingly needed to differentiate patients who are more likely to have poorer outcome^9^. Further information gained from different imaging modalities to build a more reliable prediction model for clinical practice is helpful and valuable.

As stated above, pretreatment staging is crucial. The fluorine-18 fluorodeoxyglucose (FDG), a glucose analog, can be used to measure the degree of glucose utilization. The uptake degree of FDG detected by positron emission tomography/computed tomography (PET/CT) represents the tissue metabolism on the whole-body and functional images. The FDG PET/CT has been widely used in pretreatment staging of disease and assessment of treatment response for patients diagnosed with DLBCL^10–12^. It has been reported in the literature that there are a variety of quantitative parameters deriving from image which have potential utility to predict prognosis or treatment outcome. The standardized uptake value (SUV) is the most commonly used parameter in FDG PET/CT and has been proved to be a significant prognostic predictor in DLBCL^13,14^. The percentage change in maximal SUV (SUVmax) between initial and delayed phase images is defined as the retention index (RI) which is found to have significant prognostic potential to predict overall survival (OS) in patient with DLBCL^15^. Beyond SUV and RI, the total metabolic tumor volume (MTVsum) has also been shown to be a predictor for survival outcome in many previous studies^16–19^. It was also reported that an elevated MTVsum, independent from IPI, is a predictor for shorter progression-free survival (PFS) and OS in patients with DLBCL^20^.

Learning is the process of behavior improvement over time via discovering new information. If the referred process is achieved by machine rather than human brain, it is called machine learning. The experience acquiring from the existing examples helps to find the optimal solution for coming problems in the machine learning process. Due to rapid accumulation of larger and larger raw data, the traditional methods cannot handle well and the big data concept emergences over time with the development of information technologies. Machine learning in which algorithms were used by the computers with a certain order when performing operations is a subset of artificial intelligence. Based on the training data, machine learning algorithms can search for optimal connection weights of a prespecified neural network model in order to make decisions and predictions. Some machine learning models, in which sophisticated mechanism such as high-order and non-linear interactions between predictors and the responses were used, have shown ability to improve overall clinical prediction in various conditions^21–23^.

Nonetheless, due to the heterogenous entity of DLBCL, the prognostic parameters of DLBCL, the total effects of these parameters, and individual weight of each parameter remain to be an issue deserving further research. Previous studies have reported that machine learning algorithms, using either molecular profiling data or combined clinical and genetic data, helped to do disease classification, diagnosis, and prognosis prediction^24–29^. However, there are few reports, in which using modern machine learning models and incorporating FDG PET/CT metabolic imaging parameters, to perform the outcome prediction in DLBCL.

Therefore, this study aims to develop the logistic and neural network models to predict DLBCL clinical outcome based on PFS and OS. Both clinical and metabolic parameters from FDG PET/CT scans were used as predictors. The performance of the models is also compared.

## Materials and methods

### Patient population

This is a retrospective study in which medical records of malignant lymphoma patients diagnosed at the Kaohsiung Medical University Hospital were reviewed. The Institutional Review Board of Kaohsiung Medical University Hospital approved the reviewing process of the clinical data (KMUHIRB-E(I)-20180275). Patient consent was waived because all the clinical data were retrospectively collected via the medical chart reviewing, and the waiver for subject informed consent was also approved by the Institutional Review Board of Kaohsiung Medical University Hospital. The inclusion criteria were recent diagnosis and histologically proven DLBCL. The patients had to be 18 years old or older without known previous or concurrent malignant disease. All patients underwent a complete pre-treatment work-up, including whole-body FDG PET/CT scan, bone marrow biopsy, clinical history, physical examination, and standard laboratory tests. All methods were carried out in accordance with relevant guidelines and regulations. The definite diagnosis was made by experienced pathologists based on the World Health Organization classification of lymphoid neoplasms in 2016. Only the “diffuse large B-cell lymphoma, NOS” type were enrolled. Patients’ clinical stage patient was determined with the Ann Arbor staging system and was achieved by the multi-disciplinary consensus by experts in various professional fields including radiology, nuclear medicine, pathology, and hematology.

### Treatment and clinical course

All patients received 4–6 cycles of rituximab-containing chemotherapy, that is, rituximab combined with cyclophosphamide, doxorubicin, vincristine, and prednisolone (R-CHOP), as an initial therapy. Involved field radiation therapy was administered for patients with initial clinical bulky disease or residual tumor presentation after completion of the chemotherapy. Patients experiencing refractory or relapsed disease were treated with salvage chemotherapy or received autologous stem cell transplantation (ASCT) with high-dose chemotherapy if clinically indicated. The therapeutic regimens and plans followed the National Comprehensive Cancer Network (NCCN) guidelines for B-cell lymphoma according to the year when patient was diagnosed, and the detail was decided with consensus in the multi-disciplinarily combined conference of lymphoma in accordance with the patient’s clinical condition. The PFS was defined as the time from diagnosis to disease relapse, progression or death related to lymphoma. The OS was defined as the time from diagnosis to death from any cause.

### Dataset

We collected available information (including clinical, laboratory, and metabolic imaging parameters from FDG PET/CT scans) during the pre-treatment workup as the input variables for the machine learning models. The output is a binary label regarding patient clinical outcome at the 3-year timepoint. That is, the output for PFS indicates whether the patient relapse or progresses after 3 years from the date of diagnosis; and for OS, whether the patient remained alive after 3 years from the date of diagnosis. This is a binary classification problem.

### Machine learning methodology

In this study, we used the following machine learning techniques to create prediction models: (1) logistic regression (LR), (2) random forest (RF), (3) support vector classifier (SVC), (4) deep neural network (DNN), and (5) fuzzy neural network (FNN).

First, we fitted an LR model using scikit-learn package (version 0.23.2) for Python (3.7.9). After processing, the prediction outcomes as well as the confusion matrix were computed. Second, the RF model, which is an ensemble of decision trees with bootstrapped training samples for tree induction, was adopted. In this study, the parameter setting for number of trees was 100. Third, SVC is a part of the support vector machine (SVM), which is one of the most robust prediction models. The SVC algorithm formally creates a hyperplane in which the data can be separated into two classes with the maximal soft margin. The SVC has been used in a broad range of classification and pattern recognition problems ranging from speech, text recognition, protein function prediction, and handwriting analysis^30^. So far, however, it has been rarely used in the field of cancer prediction and prognosis. Here, scikit-learn package (0.23.2) was carried to conduct the random forest model and the SVC with regularization parameter C = 1.0 and radial basis function kernel. Fourth, DNN model is a learning machine composed of multiple processing layers including many intermediate hidden layers. Each hidden unit in the hidden layer composes of the non-linear activation functions that are transformed from linear combination of predictors^31^. In the current study, four-layer feedforward model with adaptive moment estimation optimizer, rectified linear unit (Relu) activation function for hidden layers, and sigmoid activation function for output layer conducting with Keras package (2.3.1) was used. The model of DNN with architecture of 2-6-4-1 was structured and a total of 51 parameters were developed. Lastly, an FNN is intrinsically a fuzzy system represented as a neural network. Suppose the fuzzy rules in the rule base are given in the form as shown below, where *x* is the input, *y* is the output, and *A*, *B* are fuzzy sets:

- If $x_{1}$ is $A_{1j}$ and $x_{2}$ is $A_{2j}$ and … and $x_{n}$ is $A_{nj}$,
- Then $y_{1}$ is $B_{j1}$ and $y_{2}$ is $B_{j2}$ and … and $y_{p}$ is $B_{jp}$.

In this fuzzy system, we specifically use the singleton fuzzifier, product inference engine, center-average defuzzifier, and Gaussian membership functions for the fuzzy sets. Then the fuzzy system can be configured as a neural network, called an FNN. We implemented the FNN using Tensorflow (1.15.4) as a neural network layer with a total of 64 parameters. The source code for defining the FNN layer is provided in the supplementary file.

For better and reliable estimation of the predicting capability of various learning machines, we used 10-iterated fivefold cross validation (CV) with shuffling process. Fivefold CV was conducted and the whole process was then repeated 10 times, resulting in 50 testing results. Then the mean and standard deviation on the 50 testing errors was calculated to evaluate the predicting power of each model. According to the 50 testing results, the confusion matrix results were subsequently computed. The F1 values were also calculated to compare the prediction ability.

### Statistical analysis

MedCalc Statistical Software version 20.014 (MedCalc Software Ltd, Ostend, Belgium; https://www.medcalc.org; 2021) was used to perform Kaplan–Meier survival analysis, Cox proportional hazard model, and area under the curve (AUC) index for receiver operator characteristic (ROC). A *p* < 0.05 was considered statistically significant.

## Results

A total of 122 patients, 57 (33.3%) female and 65 (66.6%) males, were included in the study, with the mean age 61.3 ± 17.0 years (Table 1). Fifty-three (43.4%) patients presented as extranodal involvement at diagnosis, and 23 (18.9%) patients presented with pathologically confirmed bone marrow involvement of tumor cell. According to the Ann Arbor staging system, 16 (13.1%) cases were at stage I, 32 (26.2%) at stage II, 29 (23.8%) at stage III, and 45 (36.9%) at stage IV. As to IPI scores, patients with low risk (score 0–1), low-intermediate risk (score 2), high-intermediate risk (score 3), and high risk (score 4–5) were 39 (32.0%), 38 (31.1%), 22 (18.0%), and 23 (18.9%), respectively.

**Table 1:** Baseline characteristics at diagnosis of the 122 patients with diffuse large B-cell lymphoma.

Variable	Value (%)
Age (y)	
Mean ± SD	61.3 ± 17.0
Range	18–94
Sex	
Male	65 (53.3)
Female	57 (46.7)
Primary lesions	
Lymph nodes	69 (56.6)
Extranodal lesions	53 (43.4)
Bone marrow involvement	
Yes	23 (18.9)
No	99 (81.1)
Ann Arbor stage	
I	16 (13.1)
II	32 (26.2)
III	29 (23.8)
IV	45 (36.9)
IPI	
0–1	39 (32.0)
2	38 (31.1)
3	22 (18.0)
4–5	23 (18.9)
Hemoglobin (g/dL)	11.68 ± 1.92
WBC (× 10^3^ µL)	6.94 ± 3.10
Platelet (× 10^3^ µL)	231.7 ± 105.2
Albumin (g/dL)	3.56 ± 0.59
Creatinine (mg/dL)	0.93 ± 0.71
GOT (IU/L)	34.67 ± 24.24
GPT (IU/L)	26.39 ± 17.28
LDH (IU/L)	381.75 ± 528.10
β2-microglobulin (μg/dL)	316.80 ± 280.20
Maximal SUVt	16.12 ± 8.25
SUVb	14.36 ± 8.77
MTV (cm^3^)	664.48 ± 968.67
TLG (cm^3^)	4428.79 ± 7024.62

*SD* standard deviation, *IPI* international prognostic index, *Hb* hemoglobin, *WBC* white blood cell, *GOT* glutamate oxaloacetate transaminase, *GPT* glutamate pyruvate transaminase, *LDH* lactate dehydrogenase, *SUVt* standardized uptake value of tumor, *SUVb* maximal SUV of biopsied site, *MTV* metabolic tumor volume, *TLG* total lesion glycolysis.

Pre-treatment laboratory data were collected, including hemogram [white blood cell count (WBC), hemoglobin (Hb), and platelet count], liver function test [glutamate pyruvate transaminase (GPT), glutamate oxaloacetate transaminase (GOT)], albumin, creatinine, β2-microglobulin, and LDH. The metabolic parameters from FDG PET/CT imaging were also obtained. The mean value of maximal SUV of tumor (SUVt) and maximal SUV of biopsied site (SUVb) was 16.12 ± 8.25 and 14.36 ± 8.77 respectively. The mean values of MTVsum and total lesion glycolysis (TLG) were 664.48 ± 968.67 cm^3^ and 4428.79 ± 7024.62 cm^3^ respectively.

**Figure 1:**
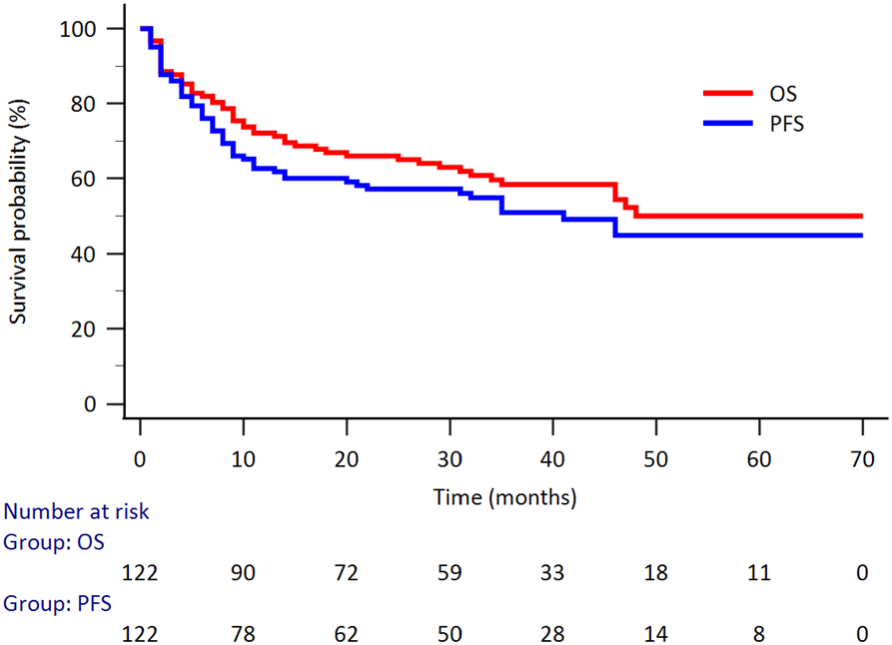
The Kaplan—Meier survival curve for the evaluation of PFS and OS in 122 patients with DLBCL. The mean PFS was 41.0 months, with the 1-year PFS 62.7%, 2-year PFS 57.2%, and 3-year PFS 51.0%, respectively. The mean OS was 43.6 months, with 1-year OS 72.1%, 3-year OS 58.4%, and 5-year OS 50.0%, respectively. The number at risk at each time point was also listed.

The survival analysis revealed the clinical outcome shows in Fig. 1. The mean PFS was 41.0 months, with the 1-year PFS 62.7%, 2-year PFS 57.2%, and 3-year PFS 51.0%, respectively. The mean OS was 43.6 months, with 1-year OS 72.1%, 3-year OS 58.4%, and 5-year OS 50.0%, respectively.

### Evaluation of PFS

**Figure 2:**
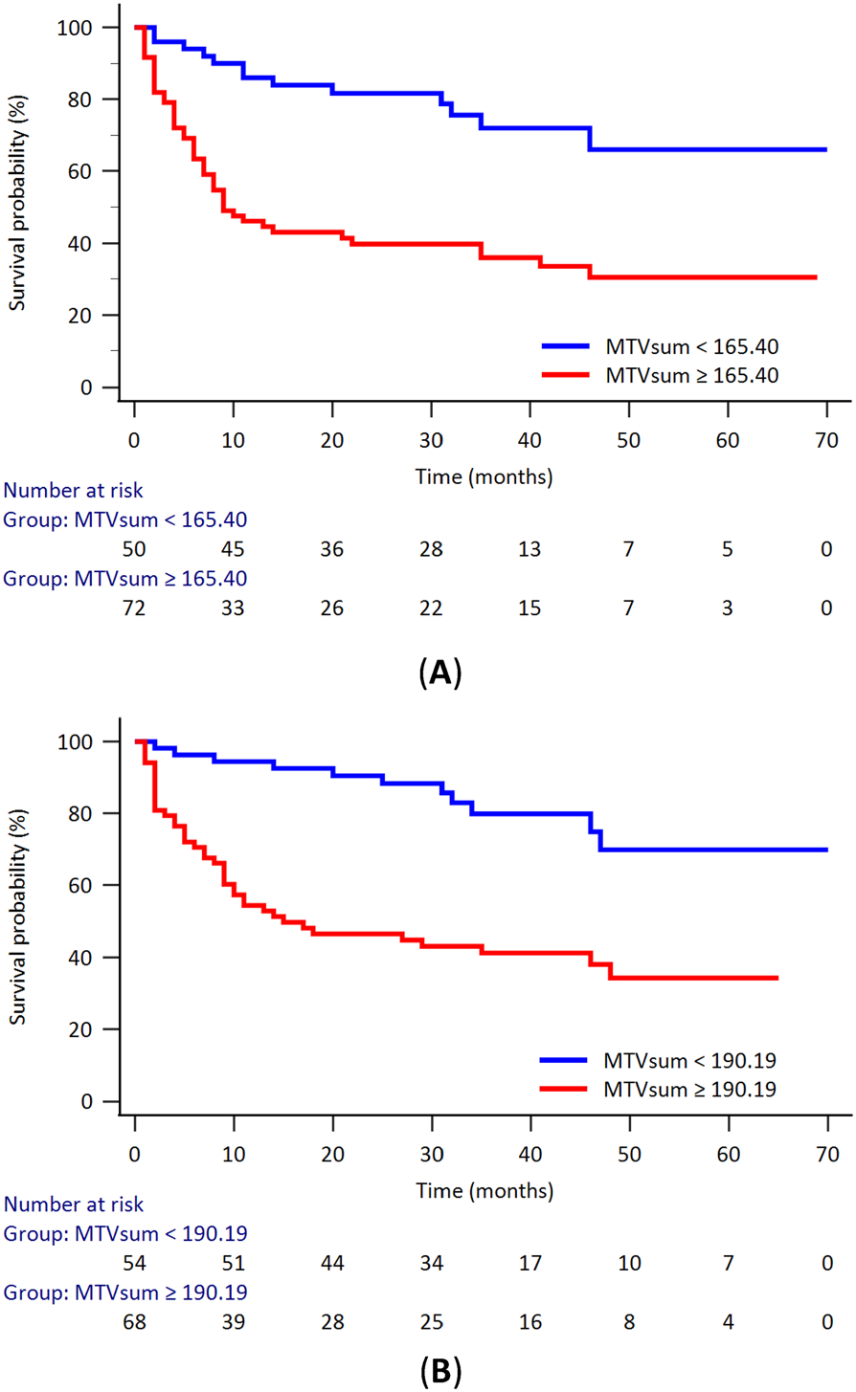
The Kaplan–Meier survival curve of the patients with DLBCL according to the MTVsum. (**A**) for the evaluation of PFS, patients with optimal cutoff value (MTVsum ≥ 165.40 cm^3^) had a poorer PFS (*p* < 0.0001); (**B**) for the evaluation of OS, patients with optimal cutoff value (MTVsum ≥ 190.19 cm^3^) had a poorer OS (*p* < 0.0001). The number at risk at each time point was also listed.

For PFS using univariate analysis, 4 variables (i.e., disease stage, IPI, LDH level, and MTVsum) revealed to be significantly correlated with the patients’ PFS. The multivariate analysis further disclosed that IPI and MTVsum were independent prognostic factors for PFS (Table 2). The cutoff values for LDH were dichotomized using normal reference. Using ROC analysis to make the dichotomization of MTVsum, the optimal cutoff value for PFS was 165.40 cm^3^ (AUC 0.78, Youden index 0.3670, *p* < 0.0001). The Kaplan–Meier survival analysis revealed patient with MTVsum ≥ 165.40 had a significantly poor PFS (log-rank *p* < 0.0001, Fig. 2A).

**Table 2:** Analysis of Cox proportional hazards models for potential prognostic factors affecting PFS.

	Univariate analysis		Multivariate analysis	
	HR (95% CI)	*p*	HR (95% CI)	*p*
Stage		0.0002*		
I	1			
II	1.59 (0.43–5.91)	0.4818		
III	4.08 (1.19–14.04)	0.0255*		
IV	5.53 (1.69–18.11)	0.0048*		
IPI		0.0001*	1.41 (1.10–1.83)	0.0089*
Low (0–1)	1			
Low-intermediate (2)	1.43 (0.67–3.06)	0.3553		
High-intermediate (3)	3.01 (1.39–6.53)	0.0053*		
High (4–5)	5.12 (2.45–10.72)	< 0.0001*		
LDH (≥ vs. < 192 IU/L))	2.84 (1.59–5.06)	0.0002*		
MTVsum (≥ vs. < 165.40 cm^3^)	3.54 (1.90–6.56)	< 0.0001*	2.00 (1.02–3.91)	0.0435*

*HR* hazard ratio, *CI* confidence interval, *IPI* international prognostic index, *LDH* lactate dehydrogenase, *MTV* metabolic tumor volume.

*Statistically significant.

For subsequent machine learning applications, we used IPI and MTVsum as the input predictors. The IPI is the international prognostic index clinically used for lymphoma, which is an integer from 0 to 5. The MTVsum is the total metabolic tumor volumes of whole-body lesions, which is the summation of whole-body tumor volumes when they showed abnormal FDG uptake. The MTVsum is a positive real number. The output is a binary label regarding patient clinical outcome (progress or not for PFS, alive or not for OS) at the 3-year timepoint.

The dataset is partitioned into training set and testing set. We use the training set to train the model. Then, we use the testing set to evaluate the performance of the model. The evaluation is based on commonly used statistical indicators given below:

$${\text{Accuracy}} = \frac{{\left( {{\text{TP}} + {\text{TN}}} \right)}}{{\left( {{\text{TP}} + {\text{FP}} + {\text{FN}} + {\text{TN}}} \right)}}$$

$${\text{Sensitivity}} = \frac{{{\text{TP}}}}{{\left( {{\text{TP}} + {\text{FN}}} \right)}}$$

$${\text{Specificity}} = \frac{{{\text{TN}}}}{{\left( {{\text{FP}} + {\text{TN}}} \right)}}$$

$${\text{PPV}} = \frac{{{\text{TP}}}}{{\left( {{\text{TP}} + {\text{FP}}} \right)}}$$

$${\text{NPV}} = \frac{{{\text{TN}}}}{{\left( {{\text{FN}} + {\text{TN}}} \right)}}$$

$${\text{F}}1 = \frac{2}{{\frac{1}{{{\text{PPV}}}} + \frac{1}{{{\text{Sensitivity}}}}}}$$

where PPV: positive predictive value; NPV: negative predictive value, where TP (true positive), FP (false positive), FN (false negative), and TN (true negative) are the 4 entries of confusion matrix of the testing results.

For fivefold CV, the whole dataset is randomly divided into 5 disjoint sets. In each iteration of the fivefold CV, one set is specified as the testing data and the rest are training set. After the training process using training data, the pre-specified metric or performance index (e.g., accuracy) is computed on the testing data. This training/testing process is performed for 5 times producing 5 testing results. The whole process is repeated 10 times constituting the so called 10-iterated fivefold CV and 50 testing results of the statistical indicators are obtained. Finally, we calculate the means and standard deviations of the 50 testing results serving as the performance of the model.

Using IPI and MTVsum as input predictors, the machine learning algorithms used namely LR, RF, SVC, DNN and FNN, had an accuracy of 0.66 ± 0.10, 0.69 ± 0.14, 0.71 ± 0.14, 0.71 ± 0.10, and 0.67 ± 0.08 respectively. The specificity was 0.74 ± 0.04, 0.76 ± 0.12, 0.84 ± 0.12, 0.77 ± 0.11, and 0.79 ± 0.14 respectively. The AUC index for ROC of these predictive models were 0.66 ± 0.10, 0.70 ± 0.13, 0.71 ± 0.13, 0.69 ± 0.09, 0.66 ± 0.10, respectively. Among these five models, SVC (0.71 ± 0.14) and DNN (0.71 ± 0.1) had the highest prediction accuracy and are statistically significant in comparison with the LR (SVC vs. LR, *p* = 0.0425; DNN vs. LR, *p* = 0.0140) and FNN (DNN vs. FNN, *p* = 0.0295) models. No statistical significance toward RF was noted (*p* = 0.4131). The results of the algorithms used are summarized in Table 3.

**Table 3:** Prediction performances of PFS in different learning machines.

	LR	RF	SVC	DNN	FNN
Accuracy	0.66 ± 0.10	0.69 ± 0.14	0.71 ± 0.14	0.71 ± 0.10	0.67 ± 0.08
Sensitivity	0.58 ± 0.22	0.65 ± 0.19	0.58 ± 0.24	0.60 ± 0.18	0.55 ± 0.16
Specificity	0.74 ± 0.04	0.76 ± 0.12	0.84 ± 0.12	0.77 ± 0.11	0.79 ± 0.14
PPV	0.63 ± 0.13	0.69 ± 0.15	0.75 ± 0.20	0.70 ± 0.13	0.66 ± 0.13
NPV	0.69 ± 0.12	0.71 ± 0.19	0.71 ± 0.14	0.70 ± 0.13	0.67 ± 0.12
F1 score	0.59 ± 0.18	0.65 ± 0.14	0.62 ± 0.22	0.62 ± 0.15	0.58 ± 0.12
AUC	0.66 ± 0.10	0.70 ± 0.13	0.71 ± 0.13	0.69 ± 0.09	0.66 ± 0.10

*LR* logistic regression, *RF* random forest, *SVC* support vector classifier, *DNN* deep neural network, *FNN* fuzzy neural network, *PPV* positive predictive value, *NPV* negative predictive, *AUC* area under curve.

### Evaluation of OS

When it comes to the OS, the univariate analysis revealed 5 variables (i.e., disease stage, IPI, LDH, albumin level, and MTVsum) were significantly correlated with the patients’ OS. Further multivariate analysis disclosed that IPI and MTVsum were independent prognostic factors for OS (Table 4). The cutoff values for albumin and LDH were dichotomized using normal reference. Using ROC analysis to make the dichotomization of MTVsum, the optimal cutoff value for OS was 190.19 cm^3^ (AUC 0.73, Youden index 0.4027, *p* < 0.0001). The Kaplan–Meier survival analysis revealed patient with MTVsum ≥ 190.19 had a significantly poor OS (log-rank *p* < 0.0001, Fig. 2B).

**Table 4:** Analysis of Cox proportional hazards models for potential prognostic factors affecting OS.

	Univariate analysis		Multivariate analysis	
	HR (95% CI)	*p*	HR (95% CI)	*p*
Stage		0.0002*		
I	1			
II	1.17 (0.30–4.54)	0.8165		
III	3.79 (1.10–13.02)	0.0343*		
IV	4.97 (1.50–16.45)	0.0086*		
IPI		< 0.0001*	2.01 (1.51–2.68)	< 0.0001*
Low (0–1)	1			
Low-intermediate (2)	3.21 (1.16–8.94)	0.0253*		
High-intermediate (3)	6.59 (2.34–18.54)	0.0004*		
High (4–5)	14.3 (5.32–38.36)	< 0.0001*		
LDH (≥ vs. < 192 IU/L))	3.37 (1.76–6.46)	0.0001*		
Albumin (< vs. ≥ 3.5 g/dL)	2.66 (1.54–4.61)	0.0005*		
MTVsum (≥ vs. < 190.19 cm^3^)	4.14 (2.12–8.07)	< 0.0001*	2.17 (1.06–4.43)	0.0335*

*HR* hazard ratio, *CI* confidence interval, *IPI* international prognostic index, *LDH* lactate dehydrogenase, *MTV* metabolic tumor volume.

*Statistically significant.

Using IPI and MTVsum as input predictors for the machine learning algorithms, the predictive accuracy showed 0.73 ± 0.05 for LR, 0.72 ± 0.12 for RF, 0.75 ± 0.13 for SVC, 0.76 ± 0.10 for DNN, and 0.75 ± 0.09 for FNN respectively (Table 5). The specificity was 0.83 ± 0.10, 0.83 ± 0.14, 0.86 ± 0.11, 0.84 ± 0.10, and 0.84 ± 0.11 respectively. The AUC index for ROC of these predictive models were 0.72 ± 0.04, 0.70 ± 0.11, 0.72 ± 0.14, 0.72 ± 0.09, 0.71 ± 0.10, respectively. The DNN model has the highest accuracy (0.76 ± 0.10) for the prediction of patient OS; however, no statistical significance was noted when compared with the other 4 models.

**Table 5:** Prediction performances of OS in different learning machines.

	LR	RF	SVC	DNN	FNN
Accuracy	0.73 ± 0.05	0.72 ± 0.12	0.75 ± 0.13	0.76 ± 0.10	0.75 ± 0.09
Sensitivity	0.60 ± 0.06	0.58 ± 0.14	0.57 ± 0.19	0.59 ± 0.19	0.58 ± 0.18
Specificity	0.83 ± 0.10	0.83 ± 0.14	0.86 ± 0.11	0.84 ± 0.10	0.84 ± 0.11
PPV	0.71 ± 0.16	0.72 ± 0.22	0.72 ± 0.18	0.75 ± 0.12	0.71 ± 0.20
NPV	0.75 ± 0.09	0.74 ± 0.12	0.75 ± 0.15	0.75 ± 0.12	0.75 ± 0.09
F1 score	0.64 ± 0.04	0.62 ± 0.14	0.63 ± 0.18	0.66 ± 0.14	0.63 ± 0.19
AUC	0.72 ± 0.04	0.70 ± 0.11	0.72 ± 0.14	0.72 ± 0.09	0.71 ± 0.10

*LR* logistic regression, *RF* random forest, *SVC* support vector classifier, *DNN* deep neural network, *FNN* fuzzy neural network, *PPV* positive predictive value, *NPV* negative predictive, *AUC* area under curve.

## Discussion

Clinically, the PET/CT scan using FDG as functional tracer has been broadly used in the management of oncologic patients for several years. It has been reported that FDG PET/CT plays clinical roles in diagnosis making, disease staging, therapeutic monitoring, and outcome prediction in patients with lymphoma^10,32–34^. Although the maximal SUV of the primary tumor has been widely demonstrated to be of prognostic values^13,14^, maximal SUV barely represents the degree of FDG uptake without presenting the volumetric concept. The volumetric analysis of MTV, which provides clinicians more information than maximal SUV, has brought increasing evidence of clinical value, especially in predicting the patient survival in several types of lymphoma^35–38^.

In DLBCL, there were also several reports mentioned the prognostic value of MTV. In stage II–III DLBCL patients^39^ and in patients with bone marrow involvement^40^, Song et al.^41^ concluded MTV was superior to Ann Arbor stage in predicting patient survival. Similar results by Sasanelli et al. disclosed that pre-treatment MTVsum is an independent predictor for clinical outcome in patients with all staging. Song et al.^42^ also reported that the MTV level is a factor to predict survival in primary gastrointestinal tract DLBCL independently. The MTV also helped to select patients with increased therapy response^43^, define a poor prognosis group and improve the predictive ability^18^ when it combined molecular characteristics or early PET/CT response.

The IPI has been used to predict prognosis in DLBCL treated with doxorubicin-containing regimens over the last 2–3 decades. This score has been validated in the rituximab era as revised IPI (R-IPI)^44^. In the current study, we also collected R-IPI as the clinical predictive parameter. When using the ROC analysis, however, the R-IPI had an AUC value inferior to that calculated by IPI, both in PFS and OS analysis. So, it was IPI rather than R-IPI used for further prediction in the machine learning models. More recently, classifications based on cells of origin and molecular characteristics allow the identifications of poor prognosis in different subtypes of patients.

As a subspecialty of artificial intelligence, machine learning denotes the development of algorithms obtaining parameters and models optimally representing the available data. There are two parts in the learning process, (1) estimating the unknown parameters from a known data set in a system, and (2) predicting new outputs of the system using the weights of these parameters. Nowadays, the amount of information to be interpreted and examined when predicting the malignant disease prognosis has been increasing rapidly. The evidence-based medicine is based on randomized controlled trials in which large patient populations and clinical data is handled. In the future, the clinical trials can be better conducted with machine learning approaches, and thus new findings can be obtained using the available data more easily. The machine learning-based clinical decision support systems, in recent years, have been emerging in dealing with certain clinical situations^21,22,45,46^. It is believed that machine learning methods become an alternative means for handling complex and large data sets and for model generation^47^.

The literature review disclosed some studies evaluating patient survival in DLBCL using machine learning models. Shipp et al. reported the successful prediction of patient OS via supervised machine learning algorithms dealing with oligonucleotide microarray gene-expression data^29^. Another paper used the hybrid machine learning approach, more specifically, both clinical and genomic date to create a single classifier to predict patient outcomes in DLBCL^27^. Ando et al.^25^ conducted the FNN model and 4 genes transcriptional profiling data to predict lymphoma survival, yielding an accuracy of 73.4% in comparison with the 68.5% accuracy using the Cox model and 17 genes. Another article by Ando et al.^26^ reported the FNN model as a powerful tool for extracting significant biological markers affecting prognosis in DLBCL. Biccler et al.^48^ collected patients from the Swedish and Danish cohorts and developed a new prognostic model based on machine learning approaches which outperformed known prognostic predictors for patients with DLBCL.

In the similar study published in 2021, Pan et al.^49^ established a tumor microenvironment (TME) related prognostic signature for DLBCL patients. When combining with IPI components, it is a promising prognostic model not only to help clarifying immune responses in the DLBCL microenvironment but also to indicate new clinical applications for immune therapy and individualized therapy in patients with DLBCL. In the current study, we incorporated both clinical (i.e., IPI) and the metabolic image parameter from FDG PET/CT scans (i.e., MTVsum) as the prognostic predictors. Additionally, both 3-year PFS and OS evaluations for 122 patients with DLBCL were performed using 5 different learning machines. For PFS, SVC and DNN had the highest survival estimation with a prediction accuracy of 71%. For OS, the DNN model has the highest accuracy for the prediction estimation (76%), and the FNN and SVC models have accuracy of 75%. The results are similar with that reported by Ando et al.^25^.

Although the current study was dealing with a relatively small patient population, we tried to develop 5 machine learning models with iterated CV method to predict clinical outcomes in patients with DLBCL. The machine learning methods may add the values for the survival prediction. It allowed clinicians to pay more attention to the follow-up and therapeutic strategies if high-risk patients were early identified. In the future, we hope that the modern predictive modeling approaches can be applied rather than barely using clinically available dichotomized variables or risk scores to predict patient survival. Further studies dealing with prospective design, larger patient population, and more specific histological subtypes based on different molecular or genetic presentations, may be conducted. Moreover, the prognostic modeling can be grounded on a combination of clinical, pathological, molecular, and metabolic imaging information.

## Conclusion

In the current study using machine learning algorithms with iterated CV method in patients with DLBCL, the best results for PFS were obtained with SVC and DNN techniques which outperform LR, RF, and FNN methods. For OS evaluation, the best results were obtained with DNN technique outperforming FNN, SVC, LR, and RF methods.

## Supplementary Information

Supplementary Information 1.

Supplementary Information 2.

## Data Availability

The datasets generated and/or analyzed during the current study are not publicly available due to further studies are ongoing, but are available from the corresponding author on reasonable request.
